# The efficiency and safety of low‐dose apatinib combined with oral vinorelbine in pretreated HER2‐negative metastatic breast cancer

**DOI:** 10.1002/cam4.7181

**Published:** 2024-04-25

**Authors:** Jia‐Yi Huang, Xue‐Lian Chen, Xiao‐Feng Xie, Lin Song, Li‐Ping Chen, Xiao‐Feng Lan, Xue Bai, Xiao Chen, Cai‐Wen Du

**Affiliations:** ^1^ Department of Medical Oncology, National Cancer Center/National Clinical Research Center for Cancer/Cancer Hospital & Shenzhen Hospital Chinese Academy of Medical Sciences and Peking Union Medical College Shenzhen 518116 Guangdong China

**Keywords:** antiangiogenesis therapy, low‐dose apatinib, metastatic breast cancer, oral vinorelbine

## Abstract

**Background:**

Apatinib is an oral small‐molecule tyrosine kinase inhibitor that blocks vascular endothelial growth factor receptor‐2. Oral vinorelbine is a semisynthetic chemotherapeutic agent of vinorelbine alkaloids. Apatinib and oral vinorelbine have been proved to be effective in the treatment of metastatic breast cancer (mBC). At present, several small sample clinical trials have explored the efficacy of apatinib combined with oral vinorelbine in the treatment of mBC.

**Methods:**

This retrospective study included 100 human epidermal growth factor receptor‐2 (HER2)‐negative mBC patients who received low‐dose apatinib (250 mg orally per day) plus oral vinorelbine until disease progression or intolerance during February 2017 and March 2023. The progression‐free survival (PFS), overall survival (OS), objective response rate (ORR), clinical benefit rate (CBR), disease control rate (DCR), and safety were analyzed by SPSS 26.0 software and GraphPad Prism 8 software. Cox proportional hazards regression model for univariate and multivariate was used to identify factors significantly related to PFS and OS.

**Results:**

The median follow‐up time for this study was 38.1 months. Among 100 patients with HER2‐negative mBC, 66 were hormone receptor (HR)‐positive/HER2‐negative and 34 were triple‐negative breast cancer (TNBC). The median PFS and OS were 6.0 months (95% CI, 5.2–6.8 months) and 23.0 months (95% CI, 19.9–26.1 months). There were no statistical differences in PFS (*p* = 0.239) and OS (*p* = 0.762) between the HR‐positive /HER2‐negative and TNBC subgroups. The ORR, CBR, and DCR were 21.0%, 58.0%, and 78.0%, respectively. Ninety‐five patients (95.0%) experienced varying grades of adverse events (AEs) and 38.0% of patients for Grades 3–4. The most common Grades 3–4 AEs that we observed were neutropenia (30.0%) and leukopenia (25.0%).

**Conclusion:**

Low‐dose apatinib combined with oral vinorelbine demonstrates potential efficacy and well tolerated for pretreated HER2‐negative mBC.

## Graphical abstract

This retrospective real world study included 100 patients with human epidermal growth factor receptor‐2‐negative metastatic breast cancer who received low‐dose apatinib (250 mg daily) in combination with oral vinorelbine. The median progression‐free survival and overall survival were 6.0 months (95% CI, 5.2–6.8 months) and 23.0 months (95% CI, 19.9–26.1 months), while the objective response rate, clinical benefit rate, and disease control rate were 21.0%, 58.0%, and 78.0%, respectively. The majority of adverse events were Grades 1–2 and the patients was well tolerated.

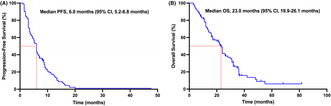

## 1 INTRODUCTION

Although the mortality of breast cancer is decreasing over the past years with the continuous development of treatment methods, metastatic breast cancer (mBC) is still incurable.^1^ The survival of human epidermal growth factor receptor‐2 (HER2)‐positive mBC has been significantly improved, which benefited by the development of anti HER2‐targeted drugs (mainly including monoclonal antibodies, tyrosine kinase inhibitors [TKIs], and antibody drug conjugates [ADCs]).^2^ HER2‐negative breast cancer includes hormone receptor (HR)‐positive/HER2‐negative breast cancer, and triple‐negative breast cancer (TNBC), accounting for 78%–85% of all breast cancer.^3^, ^4^ Endocrine therapy is often used as the preferred treatment for HR‐positive/HER2‐negative mBC, but the treatment of endocrine resistant mBC patients generally needs to refer to the treatment of TNBC.^3^ Chemotherapy is the main treatment for metastatic TNBC, but chemotherapy alone has a short remission time and is prone to drug resistance.^5^ Chemotherapy combined with immunotherapy or ADCs have achieved certain efficacy in the treatment of metastatic TNBC, however, there is no standard treatment method for metastatic TNBC with failed heavily pretreated.^6^, ^7^, ^8^

Vinorelbine is a chemotherapeutic agent of semisynthetic vinorelbine alkaloid, which inhibits tubulin polymerization and blocks mitosis in the G2‐M phase, leading to cell death.^9^, ^10^, ^11^ Compared to intravenous form, its oral form brings patients a higher quality of life and lower disease burden.^12^ Oral vinorelbine monotherapy or combined with other drugs have shown the efficacy for mBC.^13^ Previous studies reported that the objective response rate (ORR) of oral vinorelbine monotherapy in first‐line setting of mBC was 29%–31% and the median progression‐free survival (PFS) was 17.4 weeks to 5.2 months.^14^, ^15^ However, when oral vinorelbine is used in combination with other drugs in first‐line setting, the ORR reached 44.2%–51% and the PFS reached 8.4 months.^16^, ^17^ Oral vinorelbine still shows certain therapeutic efficacy in second line treatment or subsequent treatment stages.^18^, ^19^

Tumor angiogenesis plays a crucial role in the growth, invasion, and metastasis of malignant tumors.^20^ Based on this mechanism, antitumor angiogenesis therapy can be an effective means of treating malignant tumors.^21^, ^22^ The combination of antiangiogenic drug bevacizumab and chemotherapy in the treatment of mBC prolonged PFS compared to chemotherapy.^23^, ^24^, ^25^, ^26^ Some antiangiogenic small molecule TKIs have also been explored in mBC.^27^, ^28^, ^29^

Apatinib is a highly potent and selective oral small molecule TKI that blocks vascular endothelial growth factor receptor‐2 (VEGFR‐2).^30^ The clinical studies on mBC showed that apatinib monotherapy were effective with tolerable toxicity.^31^, ^32^ The combination of apatinib and chemotherapy may enhance antitumor effect through their synergistic effects,^33^ and some studies showed that apatinib combined with chemotherapy were effective for heavily pretreated mBC.^34^, ^35^ The NAN trial included 66 patients with metastatic TNBC who were randomly treated with apatinib plus vinorelbine or vinorelbine at a ratio of 1:1. The result showed that the apatinib plus vinorelbine group has a significantly longer median PFS (3.9 vs. 2.0 months, *p* = 0.026).^36^ A Phase II study enrolled 40 patients with heavily pretreated HER2‐negative mBC who were treated with apatinib plus oral vinorelbine, the median PFS was 5.2 months (95% confidence interval [CI], 3.4–7.0 months).^37^ Another clinical trial of apatinib combined with oral vinorelbine in the treatment of metastatic TNBC is in progress, and the results have not yet been reported.^38^ In the three reported studies on apatinib combined with vinorelbine in the treatment of mBC, the dose of apatinib was 500/425 mg.^36^, ^37^, ^38^ In our study, we analyzed the real‐world data to investigate the efficacy and safety of low‐dose apatinib (250 mg) combined with oral vinorelbine in pretreated HER2‐negative mBC. The advantage of our study is that the low dose of apatinib may reduce the incidence of adverse events (AEs) associated with apatinib. And we attempt to identify patient characteristics that may benefit from this all‐oral combination regimen.

## 2 PATIENTS AND METHODS

### 2.1 Study design

The present retrospective study collected the medical records of 100 patients with heavily pretreated HER2‐negative mBC at the National Cancer Center/National Clinical Research Center for Cancer/Cancer Hospital & Shenzhen Hospital, Chinese Academy of Medical Sciences and Peking Union Medical College. These patients who received low‐dose apatinib combined with oral vinorelbine during February 2017 and March 2023 were enrolled in our study. This study follows the Helsinki Declaration and has been approved by the National Cancer Hospital & Shenzhen Hospital Ethics Committee (approval number: 2022‐31‐2). As this study is a retrospective study and patient anonymization, the Ethics Committee has determined to be exempt from signing informed consent forms.

### 2.2 Patients and treatment

Eligible patients included: (1) female patients with age ≥18 years old; (2) histologically confirmed that the primary or metastatic tumor specimen was HER2‐negative breast cancer (including HR‐positive/HER2‐negative breast cancer and TNBC); (3) HER2‐negative status was defined as immunohistochemistry (IHC) 0–1+; or IHC 2+ and non‐amplification by fluorescent in situ hybridization (FISH); (4) Eastern Cooperative Oncology Group (ECOG) performance status of 0–2; (5) distant metastatic lesions diagnosed through pathological or imaging examination. Patients are excluded if (1) they had diagnosis of the second primary malignant tumor in the past 5 years; (2) had positive or undefined HER2 status; and (3) the patient is currently participating in another clinical study. All patients enrolled in our study were treated with low‐dose apatinib (250 mg orally per day, if patients presented with Grade 3/4 hematological AEs, hypertension, proteinuria, hand‐foot syndrome, or Grade 3/4 non hematological AEs that require medication intervention, delayed administration may be considered. Apatinib needs to be taken continuously until disease progression, or intolerance, or delayed for more than 21 days due to toxicity) and oral vinorelbine (take medication on the Days 1 and 8 of every 21 days, the first cycle dose for 60 mg/m^2^, and the subsequent cycles dose adjust to 80 mg/m^2^, if patients presented with Grade 3/4 hematological AEs or Grade 3/4 non hematological AEs that require medication intervention, dosage adjustment should be considered: oral vinorelbine should be reduced by 20% for the first dose and another 20% for the second dose. Oral vinorelbine needs to be taken continuously until the disease progresses or intolerance of toxicity even after dose adjustment).

### 2.3 Data collection and assessment

We collect demographic and medical data of patients by querying the medical record system, including age, ECOG score, pathological features, site of metastasis, and previous treatment. Progression‐free survival (PFS) was defined as the time from the beginning of oral administration of low‐dose apatinib plus vinorelbine to tumor progression or death from any cause; overall survival (OS) was defined as the time from the start of apatinib based treatment to death from any cause; central nervous system (CNS) response refers to the evaluation of the response of brain lesions by researchers based on brain contrast‐enhanced magnetic resonance imaging (MRI); overall response rate (ORR) was defined as the proportion of patients who got a complete response (CR) or partial response (PR); clinical benefit rate (CBR) was defined as the proportion of patients who got a CR or PR or stable disease (SD) for ≥24 weeks; disease control rate (DCR) was defined as the proportion of patients who got a CR or PR or SD. The efficacy was estimated by Response Evaluation Criteria In Solid Tumors (RECIST) version 1.1 (evaluated every two or three cycles during treatment), while toxicity was evaluated based on the 5.0 version of the Common Terminology Criteria for Adverse Events (CTCAE).

### 2.4 Statistical analyses

All statistical analyses were performed utilizing SPSS 26.0 software (IBM Corp., Armonk, NY, USA) and GraphPad Prism 8 software (GraphPad Software, Inc., La Jolla, CA, USA). The survival data (PFS and OS) were estimated by the Kaplan–Meier method, and using the Cox proportional hazards regression model for univariate and multivariate analysis to identify factors significantly related to PFS and OS. All *p*‐values and 95% CIs in this study were two‐sided. A *p*‐value < 0.05 was defined as statistically significant.

## 3 RESULTS

### 3.1 Patient characteristics

The cutoff time for follow‐up was October 31, 2023. Demographic and disease characteristics are shown in Table 1. The current study included 100 female patients with HER2‐negative mBC. Among them, there were 66 patients of HR‐positive/HER2‐negative type and 34 patients of TNBC type. The median follow‐up time for this retrospective study was 38.1 months (from 3 to 81.7 months). Seventy‐eight patients (78.0%) had an Eastern Oncology Collaborative Group (ECOG) score of 0–1, while 22 patients (22.0%) had an ECOG score of 2. In addition, 32 patients (32.0%) had Grades 1–2 histological of tumors, and 68 patients (68.0%) were Grade 3. Forty‐nine patients (49.0%) were diagnosed with Stage I–II at initial diagnosis, and 51 patients (51.0%) were diagnosed with Stage III–IV. Fifty‐five of these patients (55.0%) experienced metastasis within 2 years or had distant metastasis at initial diagnosis, while 45 patients (45.0%) metastasized to distant lesions 2 years later. The median treatment line count for the HR‐positive/HER2‐negative subgroup was four (range 2–11), while the TNBC subgroup was three (range 2–7). The majority of patients (73 out of 100, 73.0%) received at least two lines of prior systemic antitumor therapy. Seventy‐one percent (71 out of 100) of the whole participants had visceral metastases and 63 patients (63.0%) had more than three metastatic sites. Moreover, 19 patients (28.8%) received CDK4/6 inhibitors previously in the HR‐positive/HER2‐negative subgroup, and 38 patients (38.0%) received anti‐VEGFR drugs in the whole group ever before. More than 90% of patients received anthracycline and taxane treatment in the early stage or metastatic setting. Except for anthracycline and taxane, the previous chemotherapeutic agents in the metastatic setting included capecitabine (46, 46.0%), eribulin (5, 5.0%), gemcitabine (35, 35.0%), platinum (34, 34.0%), and etoposide (4, 4.0%). Among 25 patients with brain metastases, 11 patients (44.0%) had previously received brain radiotherapy, and 2 patients (8.0%) had previously received brain surgery. In summary, more than half of patients in our study had received at least 2 lines treatment previously, or with visceral metastasis, or metastasis occurring within 2 years or had more than three metastatic sites. This indicates that the patients in our study have poorer clinical and pathological characteristics, and also indicates that their treatment will be more difficult.

**TABLE 1:** Patient characteristics at baseline.

Characteristic	Total (*N* = 100; *n*, %)	HR‐positive/HER2‐negative (*N* = 66; *n*, %)	TNBC (*N* = 34; *n*, %)
Age, years			
<50	72 (72.0)	48 (72.7)	24 (70.6)
≥50	28 (28.0)	18 (27.3)	10 (29.4)
Location			
Left	54 (54.0)	38 (57.6)	16 (47.1)
Right	42 (42.0)	26 (39.4)	16 (47.1)
Bilateral	4 (4.0)	2 (3.0)	2 (5.8)
ECOG performance status			
0–1	78 (78.0)	50 (75.8)	28 (82.4)
2	22 (22.0)	16 (24.2)	6 (17.6)
Histopathologic grade			
I–II	32 (32.0)	24 (36.4)	8 (23.5)
III	68 (68.0)	42 (63.6)	26 (76.5)
TNM stage at diagnosis			
I–II	49 (49.0)	31 (47.0)	18 (52.9)
III–IV	51 (51.0)	35 (53.0)	16 (47.1)
DFS duration, months			
≤24	55 (55.0)	32 (48.5)	23 (67.6)
>24	45 (45.0)	34 (51.5)	11 (32.4)
Lines of treatment, lines			
2	27 (27.0)	12 (18.2)	15 (44.1)
≥3	73 (73.0)	54 (81.8)	19 (55.9)
Type of metastatic site			
Non‐visceral	29 (29.0)	18 (27.3)	11 (32.4)
Visceral	71 (71.0)	48 (72.7)	23 (67.6)
Number of metastatic sites, *n*			
≤3	37 (37.0)	26 (39.4)	11 (32.4)
>3	63 (63.0)	40 (60.6)	23 (67.6)
Metastatic sites			
Bone	59 (59.0)	47 (71.2)	12 (35.3)
Lung	54 (54.0)	32 (48.5)	22 (64.7)
Liver	45 (45.0)	34 (51.5)	11 (32.4)
Brain	25 (25.0)	18 (27.3)	7 (20.6)
Prior target treatment			
CDK4/6 inhibitors	19 (19.0)	19 (28.8)	0 (0.0)
Anti‐VEGFR treatment	38 (38.0)	23 (34.8)	15 (44.1)
Previous chemotherapy			
Anthracycline	90 (90.0)	60 (90.9)	30 (88.2)
Taxane	96 (96.0)	64 (97.0)	32 (94.1)
Capecitabine	46 (46.0)	29 (43.9)	17 (50.0)
Eribulin	5 (5.0)	4 (6.1)	1 (2.9)
Gemcitabine	35 (35.0)	23 (34.8)	12 (35.3)
Platinum	34 (34.0)	18 (27.3)	16 (47.1)
Etoposide	4 (4.0)	4 (6.1)	0 (0.0)
Prior local treatment (patients with brain metastases)	*N* = 25	*N* = 18	*N* = 7
Brain radiotherapy	11 (44.0)	7 (38.9)	4 (57.1)
Brain surgery	2 (8.0)	2 (11.1)	0 (0.0)

Abbreviations: CDK4/6, cyclin‐dependent kinase 4/6; DFS, disease‐free survival; ECOG, Eastern Cooperative Oncology Group; HER2, human epidermal growth factor receptor‐2; HR, hormone receptor; TNBC, triple‐negative breast cancer; TNM stage, the stage of tumor, node and metastasis; VEGFR, vascular endothelial growth factor receptor.

### 3.2 Outcomes

**FIGURE 1:**
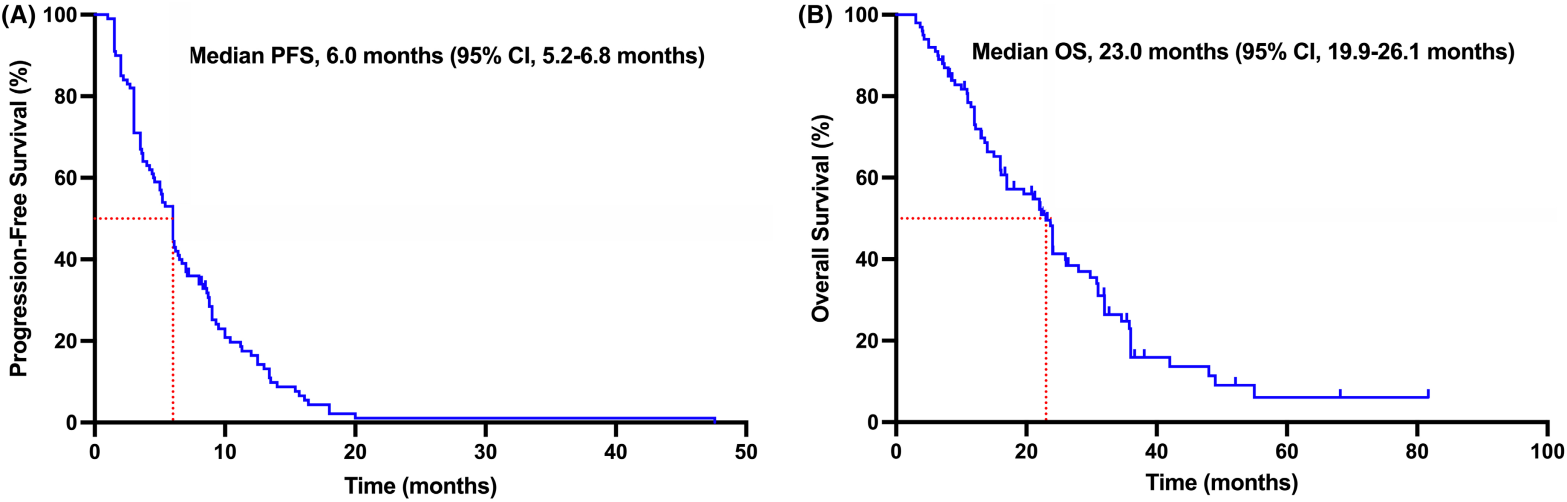
PFS curve (A) and OS curve (B) of HER2‐negative metastatic breast cancer patients treated with low‐dose apatinib plus oral vinorelbine. CI, confidence interval; HER2, human epidermal growth factor receptor‐2; OS, overall survival; PFS, progression‐free survival.

**FIGURE 2:**
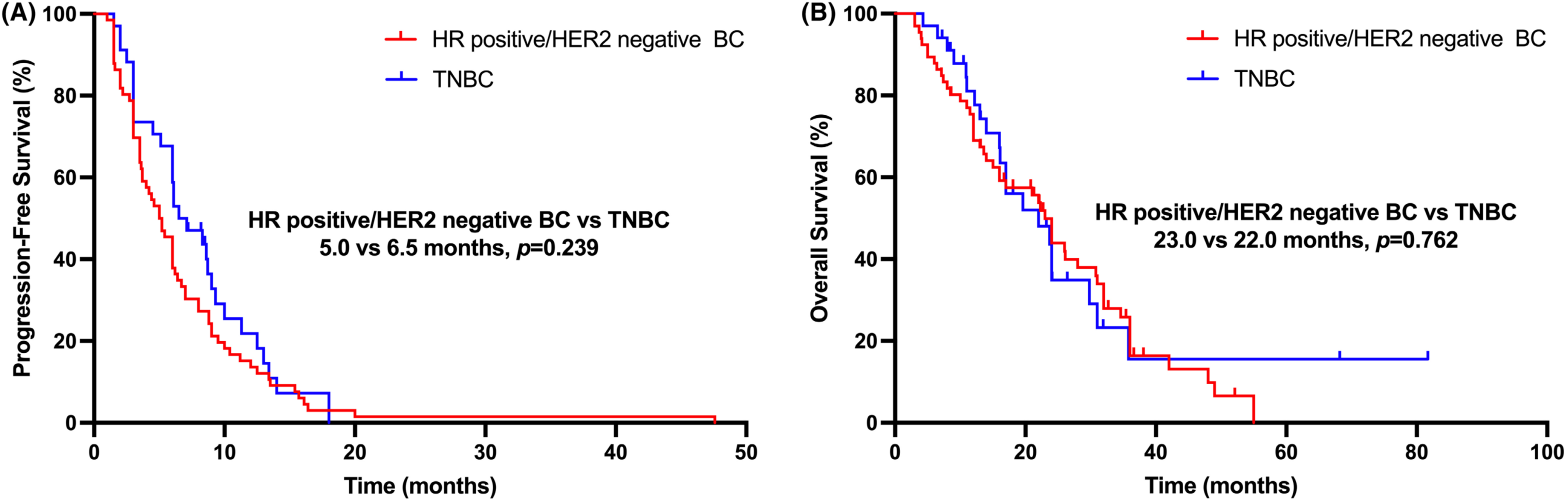
Survival curves in HER2‐negative metastatic breast cancer patients treated with low‐dose apatinib plus oral vinorelbine. (A) PFS curves of patients for HR‐positive/HER2‐negative and TNBC subgroups; (B) OS curves of patients for HR‐positive/HER2‐negative and TNBC subgroups. BC, breast cancer; HER2, human epidermal growth factor receptor‐2; HR, hormone receptor; OS, overall survival; PFS, progression‐free survival; TNBC, triple‐negative breast cancer.

As of the last follow‐up date, 3 patients were still receiving low‐dose apatinib combined with oral vinorelbine, and 28 patients were still alive. As shown in Table 2 and Figure 1, the median PFS was 6.0 months (95% CI, 5.2–6.8 months) and the median OS was 23.0 months (95% CI, 19.9–26.1 months). In the HR‐positive/HER2‐negative subgroup, the median PFS was 5.0 months (95% CI, 3.8–6.2 months) and the median OS was 23.0 months (95% CI, 19.8–26.2 months). In the TNBC subgroup, the median PFS was 6.5 months (95% CI, 3.9–9.1 months) and the median OS was 22.0 months (95% CI, 11.9–32.1 months). There was no statistical difference in PFS (5.0 vs. 6.5 months, *p* = 0.239) between the HR‐positive/HER2‐negative and TNBC subgroups (seen in Figure 2A). There was also no statistically difference in OS (HR‐positive/HER2‐negative vs. TNBC, 23.0 vs. 22.0 months, *p* = 0.762) between these two subgroups (seen in Figure 2B).

**TABLE 2:** Response to apatinib plus oral vinorelbine for HER2‐negative breast cancer patients.

	Total (*n* = 100)	HR‐positive/HER2‐negative (*n* = 66)	TNBC (*n* = 34)
PFS (months)	6.0	5.0	6.5
95% CI	(5.2–6.8)	(3.8–6.2)	(3.9–9.1)
OS (months)	23.0	23.0	22.0
95% CI	(19.9–26.1)	(19.8–26.2)	(11.9–32.1)
ORR (%)	21.0	19.7	23.5
95% CI	(12.9–29.1)	(9.8–29.5)	(8.5–38.6)
CBR (%)	58.0	53.0	67.6
95% CI	(48.2–67.8)	(40.7–65.4)	(51.1–84.2)
DCR (%)	78.0	75.8	82.4
95% CI	(69.7–89.3)	(65.1–86.4)	(68.9–95.9)

Abbreviations: CBR, clinical benefit rate; CI, confidence interval; DCR, disease control rate; HER2, human epidermal growth factor receptor‐2; HR, hormone receptor; ORR, overall response rate; OS, overall survival; PFS, progression‐free survival; TNBC, triple‐negative breast cancer.

The ORR was 21.0% (95%CI, 12.9%–29.1%) in the whole group, while the ORR were 19.7% (95%CI, 9.8%–29.5%) in the HR‐positive/HER2‐negative subgroup and 23.5% (95%CI, 8.5% to 38.6%) in the TNBC subgroup. The CBR the whole group was 58.0% (58 patients who achieved CR, PR, or SD for ≥24 week), and the DCR was 78.0% (78 patients who achieved CR, PR, or SD) (seen in Table 2).

As shown in Table 3, univariate analysis showed that patients initially diagnosed with Stage III–IV diseases (*p* = 0.002), DFS ≤24 months (*p* = 0.021), and received third‐line or above treatment (*p* = 0.003) may be more likely to progress from this combination therapy. Univariate analysis of OS showed that high‐risk factors that may promote death include DFS ≤24 months (*p* = 0.031), received third‐line or above treatment (*p* = 0.008), with liver metastases (*p* = 0.033), and with brain metastases (*p* = 0.006).

**TABLE 3:** Cox univariate regression analysis for progression‐free survival and overall survival.

Variable	Progression‐free survival			Overall survival		
	HR	95% CI	*p*‐Value	HR	95% CI	*p*‐Value
Age, years (<50 vs. ≥50)	0.999	0.639–1.560	0.995	1.382	0.822–2.325	0.223
ECOG (0–1 vs. 2)	0.715	0.435–1.176	0.187	0.701	0.410–1.200	0.196
Histopathologic grade (1–2 vs. 3)	1.062	0.691–1.632	0.784	0.836	0.505–1.384	0.485
TNM stage at diagnosis (I–II vs. III–IV)	0.514	0.339–0.778	**0.002**	0.665	0.416–1.063	0.088
DFS duration, months (≤24 vs. >24)	1.637	1.078–2.485	**0.021**	1.722	1.051–2.824	**0.031**
Lines of treatment, lines (2 vs. ≥3)	0.499	0.314–0.792	**0.003**	0.450	0.250–0.810	**0.008**
Number of metastatic sites (≤3 vs. >3)	0.970	0.640–1.470	0.885	0.627	0.376–1.045	0.073
Visceral metastases (no vs. yes)	0.763	0.491–1.186	0.229	0.765	0.452–1.295	0.319
Liver metastases (no vs. yes)	0.669	0.446–1.004	0.052	0.604	0.380–0.961	**0.033**
Lung metastases (no vs. yes)	0.907	0.604–1.361	0.636	0.893	0.560–1.427	0.637
Bone metastases (no vs. yes)	0.857	0.568–1.291	0.459	0.849	0.531–1.359	0.496
Brain metastases (no vs. yes)	0.693	0.435–1.103	0.122	0.490	0.294–0.817	**0.006**

Abbreviations: CI, confidence interval; DFS, disease‐free survival; ECOG, Eastern Cooperative Oncology Group; HR, hazard ratio; TNM stage, the stage of tumor, node and metastasis

Bold values represent p values < 0.05.

**FIGURE 3:**
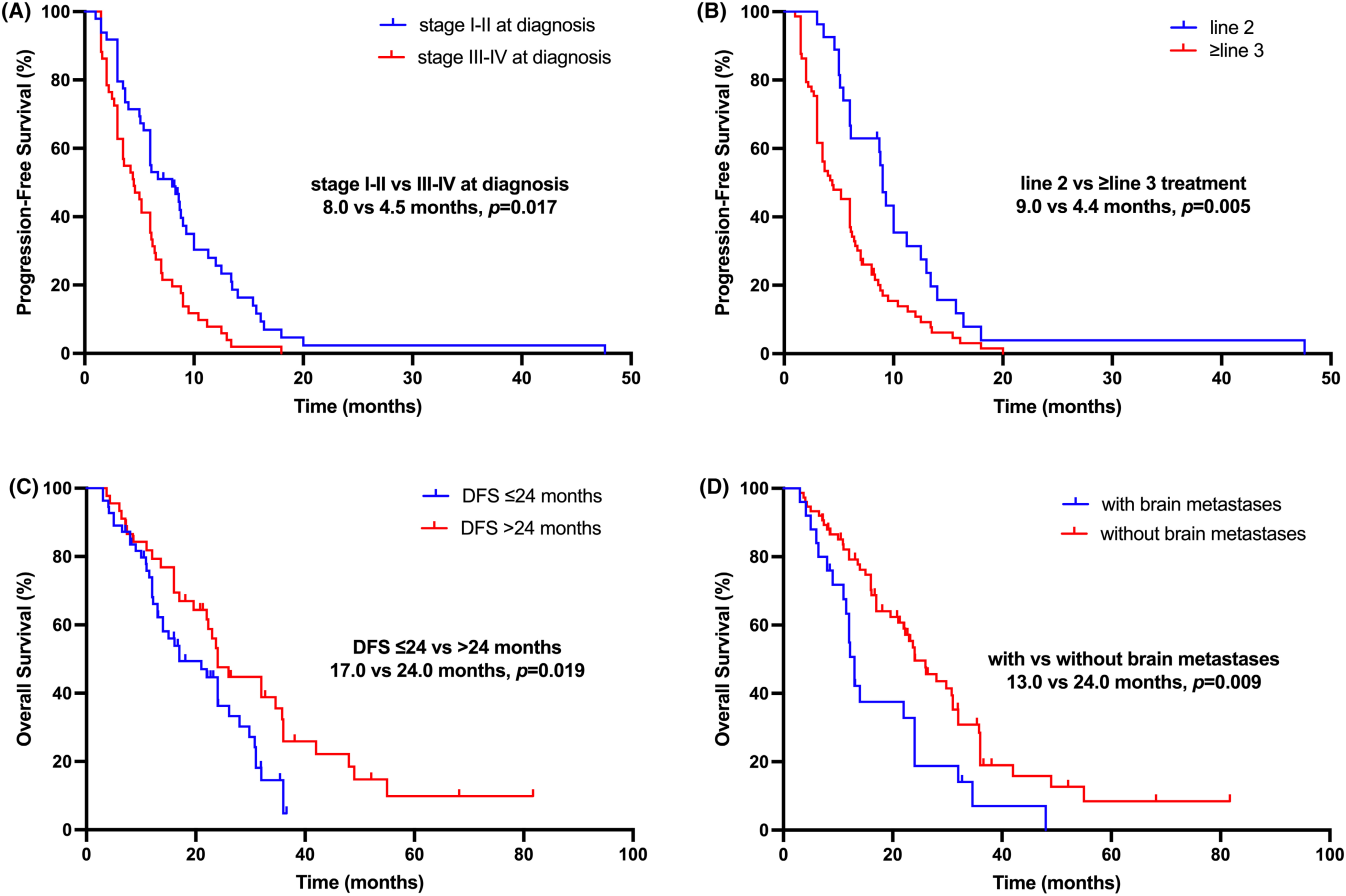
Survival curves in HER2‐negative metastatic breast cancer patients treated with low‐dose apatinib plus oral vinorelbine. (A) PFS curves of patients for initial diagnosed with Stage I–II and Stage III–IV subgroups; (B) PFS curves of patients treated for second line and third‐line or above subgroups; (C) OS curves of patients whose DFS ≤24 and >24 months' subgroups; (D) OS curves of patients with and without brain metastases subgroups. DFS, disease‐free survival; HER2, human epidermal growth factor receptor‐2; OS, overall survival; PFS, progression‐free survival.

In addition, we conducted a multivariate analysis of three factors that affect PFS in univariate analysis and found that patients initial diagnosed with Stage III–IV diseases (Stage I–II vs. III–IV, 8.0 vs. 4.5 months, HR = 0.592, *p* = 0.017) and received third‐line or above treatment (Line 2 vs. ≥Line 3, 9.0 vs. 4.4 months, HR = 0.518, *p* = 0.005) would lead to a shorter PFS. A multivariate analysis was conducted on the four factors influencing OS in univariate analysis, and it was found that patients whose DFS ≤24 months (DFS ≤24 vs. >24 months, 17.0 vs. 24.0 months, HR = 1.853, *p* = 0.019) and with brain metastases (without vs. with brain metastases, 24.0 vs. 13.0 months, HR = 0.485, *p* = 0.009) would lead to a shorter OS (seen in Table 4; Figure 3).

**TABLE 4:** Cox multivariate regression analysis for progression‐free survival and overall survival.

Variable	Progression‐free survival			Overall survival		
	HR	95% CI	*p*‐Value	HR	95% CI	*p*‐Value
TNM stage at diagnosis (I–II vs. III–IV)	0.592	0.386–0.909	**0.017**	‐	‐	‐
DFS duration, months (≤24 vs. >24)	1.467	0.949–2.266	0.085	1.853	1.109–3.098	**0.019**
Lines of treatment, lines (2 vs. ≥3)	0.518	0.326–0.824	**0.005**	0.585	0.315–1.086	0.090
Liver metastases (no vs. yes)	‐	‐	‐	0.732	0.451–1.187	0.206
Brain metastases (no vs. yes)	‐	‐	‐	0.485	0.283–0.832	**0.009**

Abbreviations: CI, confidence interval; DFS, disease‐free survival; HR, hazard ratio; TNM stage, the stage of tumor, node and metastasis.

Bold values represent p values < 0.05

Our study included 25 patients with brain metastases from breast cancer (including 18 patients with HR‐positive/HER2‐negative and 7 patients with TNBC). The results showed that the PFS and OS of the total population were 4.5 months and 13.0 months, the PFS and OS of the HR‐positive/HER2‐negative subgroup were 4.4 months and 12.0 months, and the PFS and OS of the TNBC subgroup were 4.5 months and 13.0 months, respectively.

### 3.3 Safety

All included patients received at least two cycles of treatment with low‐dose apatinib plus oral vinorelbine, and their safety was assessed. The most common AEs reported in previous studies on the treatment of apatinib or oral vinorelbine were collected. Table 5 summarized the AEs at any grade in our retrospective study. Among 100 patients included in this study, 95 patients (95.0%) experienced varying grades of AEs, with an incidence rate of 38.0% for Grades 3–4 AEs. The common AEs of any grade were leukopenia (72.0%), neutropenia (68.0%), diarrhea (58.0%), secondary hypertension (54.0%), vomiting (52.0%), nausea (51.0%), hand‐foot syndrome (49.0%), proteinuria (45.0%), aspartate aminotransferase increase (45.0%), anemia (41.0%), and alanine aminotransferase increase (39.0%). In addition, Grades 3–4 AEs were neutropenia (30.0%), leukopenia (25.0%), secondary hypertension (4.0%), alanine aminotransferase increase (3.0%), diarrhea (2.0%), hand‐foot syndrome (2.0%), proteinuria (2.0%), anemia (2.0%), aspartate aminotransferase increase (1.0%), and thrombocytopenia (1.0%). Two patients had Grade 1 bleeding (one was bleeding gums and another was nasal bleeding), which did not lead to withdrawal. Eight patients stopped treatment due to AEs during the treatment period, of which 6 patients were retreated with low‐dose apatinib plus oral vinorelbine after adjusting the dose of oral vinorelbine.

**TABLE 5:** Adverse events.

Adverse events	All grade, *n* (%)	≥Grade 3, *n* (%)
Leukopenia	72 (72.0)	25 (25.0)
Neutropenia	68 (68.0)	30 (30.0)
Diarrhea	58 (58.0)	2 (2.0)
Secondary hypertension	54 (54.0)	4 (4.0)
Vomiting	52 (52.0)	0 (0.0)
Nausea	51 (51.0)	0 (0.0)
Hand‐foot syndrome	49 (49.0)	2 (2.0)
Proteinuria	45 (45.0)	2 (2.0)
Aspartate aminotransferase increase	45 (45.0)	1 (1.0)
Anemia	41 (41.0)	2 (2.0)
Alanine aminotransferase increase	39 (39.0)	3 (3.0)
Hypercholesterolemia	19 (19.0)	0 (0.0)
Hypertriglyceridemia	16 (16.0)	0 (0.0)
Thrombocytopenia	15 (15.0)	1 (1.0)
Oral mucositis	8 (8.0)	0 (0.0)
Hemorrhage	2 (2.0)	0 (0.0)

## 4 DISCUSSION

This retrospective study explored the efficacy and safety of low‐dose apatinib combined with oral vinorelbine in the treatment of pretreated mBC. In this study, more than half of the patients (73 out of 100, 73.0%) had previously received at least two lines treatment in metastatic setting. The median PFS and OS of all 100 patients were 6.0 months (95% CI, 5.2–6.8 months) and 23.0 months (95% CI, 19.9–26.1 months), respectively. In addition, the ORR, CBR and DCR of this retrospective study were 21.0% (21 out of 100), 58.0% (58 out of 100), and 78.0% (78 out of 100). The results of this study indicate that low‐dose apatinib combined with oral vinorelbine may exhibit potential antitumor activity in pretreated HER2‐negative mBC.

Although targeted therapy and biological immunotherapy have made great progress in recent years, chemotherapy is still one of the important treatment methods for mBC. Anthracyclines and/or taxanes are often used for (neo)adjuvant treatment in early stage or first‐line treatment in metastatic setting, while there are no standards for selecting chemotherapy drugs for second‐line treatment or above.^39^ Oral vinorelbine exhibits antitumor activity not only in the first line but also in the patients previously treated with anthracyclines and taxanes.^13^ Previous studies have shown that the PFS of vinorelbine combined with capecitabine for second‐line treatment of mBC was 3.4 to 3.8 months, and the OS was 11.3 months.^18^, ^40^ From this, the efficacy of chemotherapy alone (such as oral vinorelbine combined with capecitabine) in second‐line or above treatment does not seem satisfactory. In the second‐line treatment of metastatic TNBC, sacituzumab govitecan (SG) significantly prolonged PFS (5.7 vs. 1.5 months, HR: 0.41) compared with chemotherapy of physician's choice (TPC).^41^ In heavily pretreated HR‐positive/HER2‐negative mBC patients, SG also significantly prolonged PFS compared with TPC (5.5 vs. 4.0 months, HR: 0.66).^42^ In patients with HER2‐low advanced breast cancer, trastuzumab deruxtecan (T‐Dxd) shows more encouraging efficacy (T‐Dxd group vs. TPC group: 9.9 vs. 5.1 months, HR: 0.50).^8^ The emergence of these ADCs has brought new hope for HER2‐negative mBC in later‐line settings, however, few patients are able to receive these treatments due to their high prices.

Two clinical trials reported the PFS of apatinib monotherapy in the treatment of metastatic TNBC and non TNBC were 3.3 months and 4 months, respectively, with an ORR of 10.7% and 16.7%.^31^, ^32^ A meta‐analysis showed that the ORR of apatinib monotherapy for mBC was 20.4%, and the PFS was 4 months.^43^ Therefore, apatinib monotherapy has certain antitumor activity in mBC. When apatinib combined with chemotherapy, the efficacy seems to be better. The ORR of apatinib combined with chemotherapy for mBC was 22.7%–35.5%, and the PFS was 4.7 months to 6.9 months.^44^, ^45^, ^46^ A Phase II clinical trial about apatinib combined with oral vinorelbine for HER2‐negative mBC demonstrated that the ORR was 17.1%, the PFS was 5.2 months and the OS was 17.4 months.^37^ Compared to these results, the PFS and OS in our study were both longer and had a higher ORR. As a second‐line treatment, the median PFS of our study (9 months) also appears to be longer than the Phase II clinical study reported by Zhu et al. (6.1 months).^37^ The NAN trial results showed a median PFS of 3.9 months, an OS of 11.5 months, and an ORR of 9.1% for the treatment of metastatic TNBC treated with apatinib plus vinorelbine.^36^ In the TNBC subgroup in our study, the median PFS, OS, and ORR were 6.5 months, 22 months, and 23.5%, respectively, which seemed to be better than the results of the NAN study.

In addition, we analyzed the factors that affect PFS and OS, and found that patients who were initially diagnosed in Stages III–IV or received as a third‐line treatment or beyond had a shorter PFS, while patients with DFS ≤24 months or with brain metastases had a shorter OS. Another real‐world study of low‐dose apatinib plus chemotherapy for mBC also showed that patients treated as third‐line or above had worse PFS (treated as third‐line or above vs. first‐ or second‐line: 3.5 vs. 5.1 months, *p* = 0.034).^44^ In the Phase II clinical study reported by Zhu et al., the PFS of patients treated ≥3 lines (5.2 months) was also shorter than that of patients treated <3 lines (6.1 months), but there was no statistically significant difference between the two group (*p* = 0.875).^37^ Our research findings indicated that patients with second‐line treatment or initially diagnosed with Stages I–II have longer disease remission time, while patients who experience metastasis >24 months or without brain metastases have longer survival times. This discovery may help choose patients who would benefit from this all oral combination therapy.

In terms of safety, the AE incidence rate calculated in this study was similar to previous reports,^37^, ^47^ and no new AEs were found. The majority of AEs were Grades 1–2, and the incidences of Grades 3–4 AEs were low, while most AEs can be relieved through dose reduction or symptomatic treatment. The any grade AEs with an incidence rate >40% in our study were myelosuppression, gastrointestinal reaction, secondary hypertension, hand‐foot syndrome and proteinuria. The most common Grades 3–4 AEs that we observed were neutropenia and leukopenia. We found that compared to previous reports, the incidence of secondary hypertension (4%), hand‐foot syndrome (2%) and proteinuria (2%) in Grades 3–4 in our study was lower, which were related to our use of low‐dose apatinib. Based on these research results, it can be concluded that the antitumor activity of low‐dose apatinib is not inferior to that of conventional dose, and its side effects and economic burden are lighter, making it a treatment option.

This is a retrospective study to evaluate the efficacy of low‐dose apatinib combined with oral vinorelbine in the treatment of mBC. Due to limitations such as retrospective design, small sample size, lack of randomized controlled group, and confounding factors, bias may inevitably arise. As a result of these biases, the results of this study cannot be the basis for the treatment of all HER2‐negative mBC. In the future, it needs to be confirm the efficacy of this all‐oral combination regimen through large‐scale prospective randomized controlled clinical trials. However, these results show that low‐dose apatinib combined with oral vinorelbine can effectively treat HER2‐negative mBC with acceptable toxicity, which provides an alternative treatment for such patients.

## 5 CONCLUSIONS

Finally, the findings of this retrospective study showed that low‐dose apatinib combined with oral vinorelbine exhibits potential efficacy for pretreated HER2‐negative mBC. Moreover, the regimen based on low‐dose apatinib has mild adverse reactions and well tolerated.

## AUTHOR CONTRIBUTIONS

**Jia‐Yi Huang:** Data curation (lead); formal analysis (lead); writing – original draft (lead). **Cai‐Wen Du:** Conceptualization (lead); writing – review and editing (lead). **Xue‐Lian Chen:** Investigation (equal). **Xiao‐Feng Xie:** Investigation (equal). **Lin Song:** Investigation (equal). **Li‐Ping Chen:** Investigation (equal). **Xiao‐Feng Lan:** Investigation (equal). **Xue Bai:** Investigation (equal). **Xiao Chen:** Investigation (equal).

## FUNDING INFORMATION

This work was supported by Shenzhen High‐level Hospital Construction Fund, Shenzhen Key Medical Discipline Construction Fund (No. SZXK013) and the National Natural Science Foundation of China (No. 81671750, 2016).

## CONFLICT OF INTEREST STATEMENT

The authors declare that they have no competing interest to report regarding the present study.

## ETHICS STATEMENT

This study follows the Helsinki Declaration and has been approved by the National Cancer Hospital & Shenzhen Hospital Ethics Committee (approval number: 2022‐31‐2). As this study is a retrospective study and patient anonymization, the Ethics Committee has determined to be exempt from signing informed consent forms.

## Data Availability

The datasets generated during and/or analyzed during the current study are available from the corresponding author on reasonable request.
